# Supplementary material for: Comprehensive Analysis of Imipenemase (IMP)-Type Metallo-β-Lactamase: A Global Distribution Threatening Asia
Source: Antibiotics (Basel). 2022 Feb 11;11(2):236. doi: 10.3390/antibiotics11020236 (PMC8868347; doi:10.3390/antibiotics11020236)
Supplement: Supplementary file 1 [file antibiotics-11-00236-s001.zip › antibiotics-1569229-SI.pdf]

Supplementary Materials

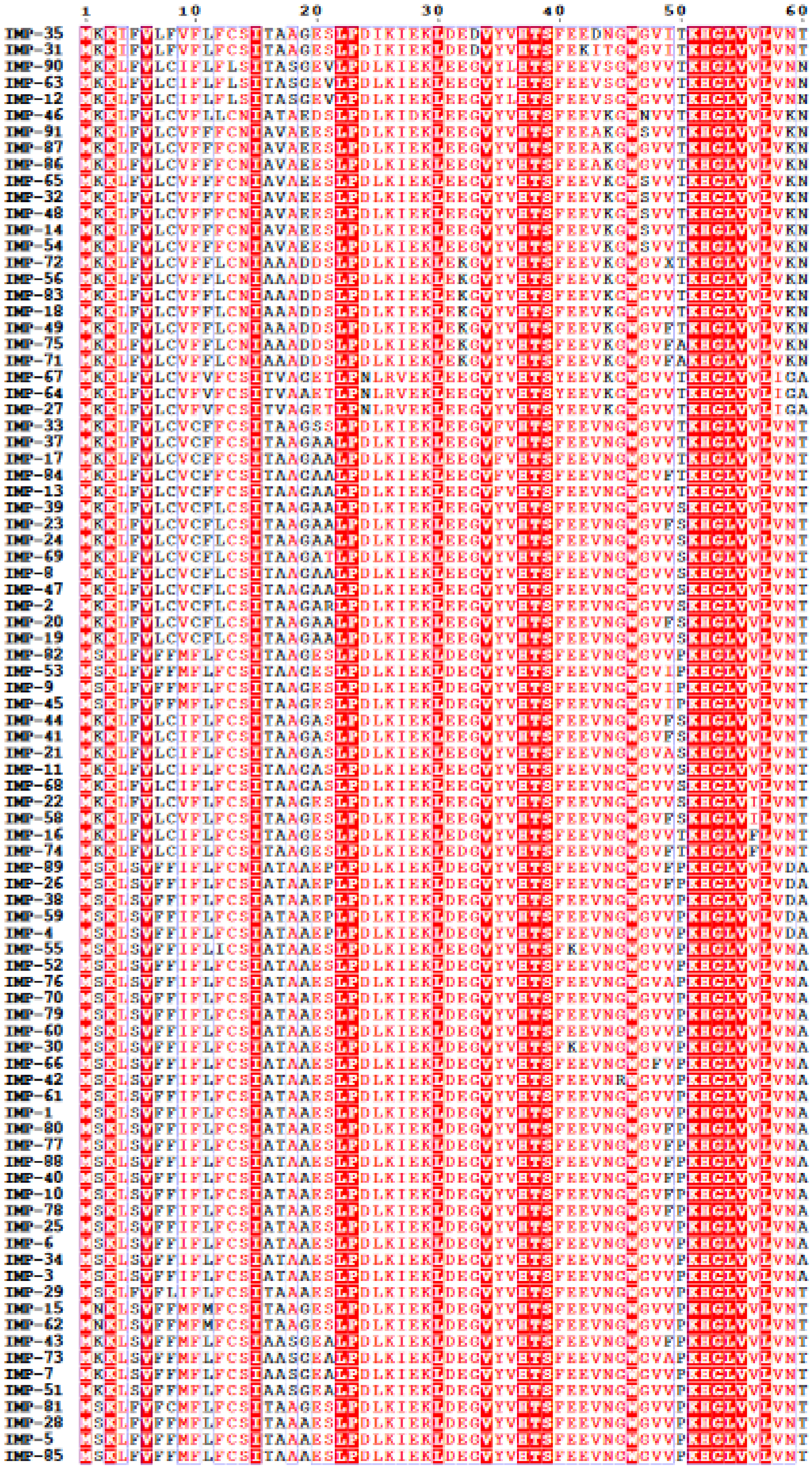

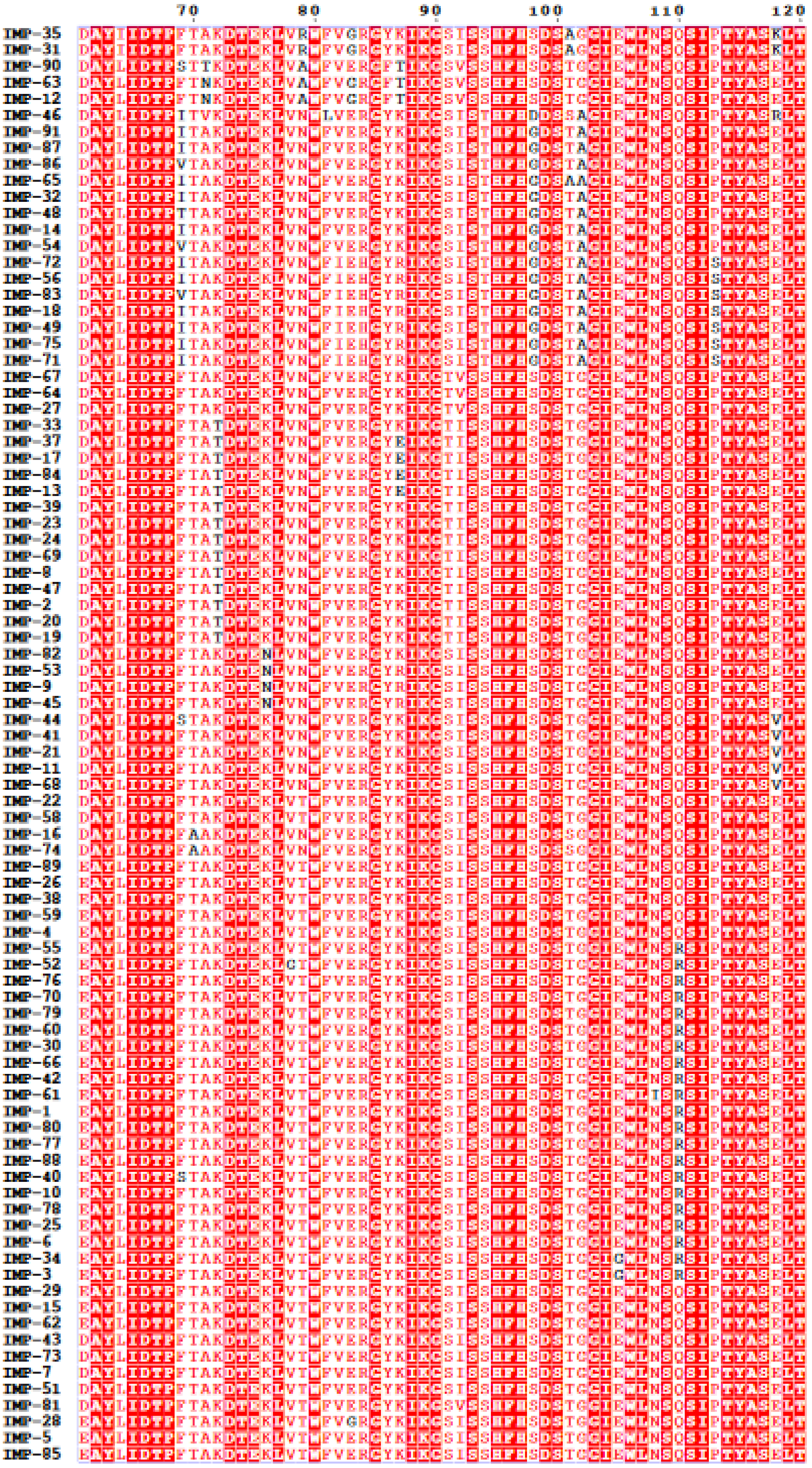

70
80
90
100
110
120
IMP-35
IMP-31
IMP-90
IMP-63
IMP-12
IMP-46
IMP-91
IMP-87
IMP-86
IMP-65
IMP-32
IMP-48
IMP-14
IMP-54
IMP-72
IMP-56
IMP-83
IMP-18
IMP-49
IMP-75
IMP-71
IMP-67
IMP-64
IMP-27
IMP-33
IMP-37
IMP-17
IMP-84
IMP-13
IMP-39
IMP-23
IMP-24
IMP-69
IMP-8
IMP-47
IMP-2
IMP-20
IMP-19
IMP-82
IMP-53
IMP-9
IMP-45
IMP-44
IMP-41
IMP-21
IMP-11
IMP-68
IMP-22
IMP-58
IMP-16
IMP-74
IMP-89
IMP-26
IMP-38
IMP-59
IMP-4
IMP-55
IMP-52
IMP-76
IMP-70
IMP-79
IMP-60
IMP-30
IMP-66
IMP-42
IMP-61
IMP-1
IMP-80
IMP-77
IMP-88
IMP-40
IMP-10
IMP-78
IMP-25
IMP-6
IMP-34
IMP-3
IMP-29
IMP-15
IMP-62
IMP-43
IMP-73
IMP-7
IMP-51
IMP-81
IMP-28
IMP-5
IMP-85

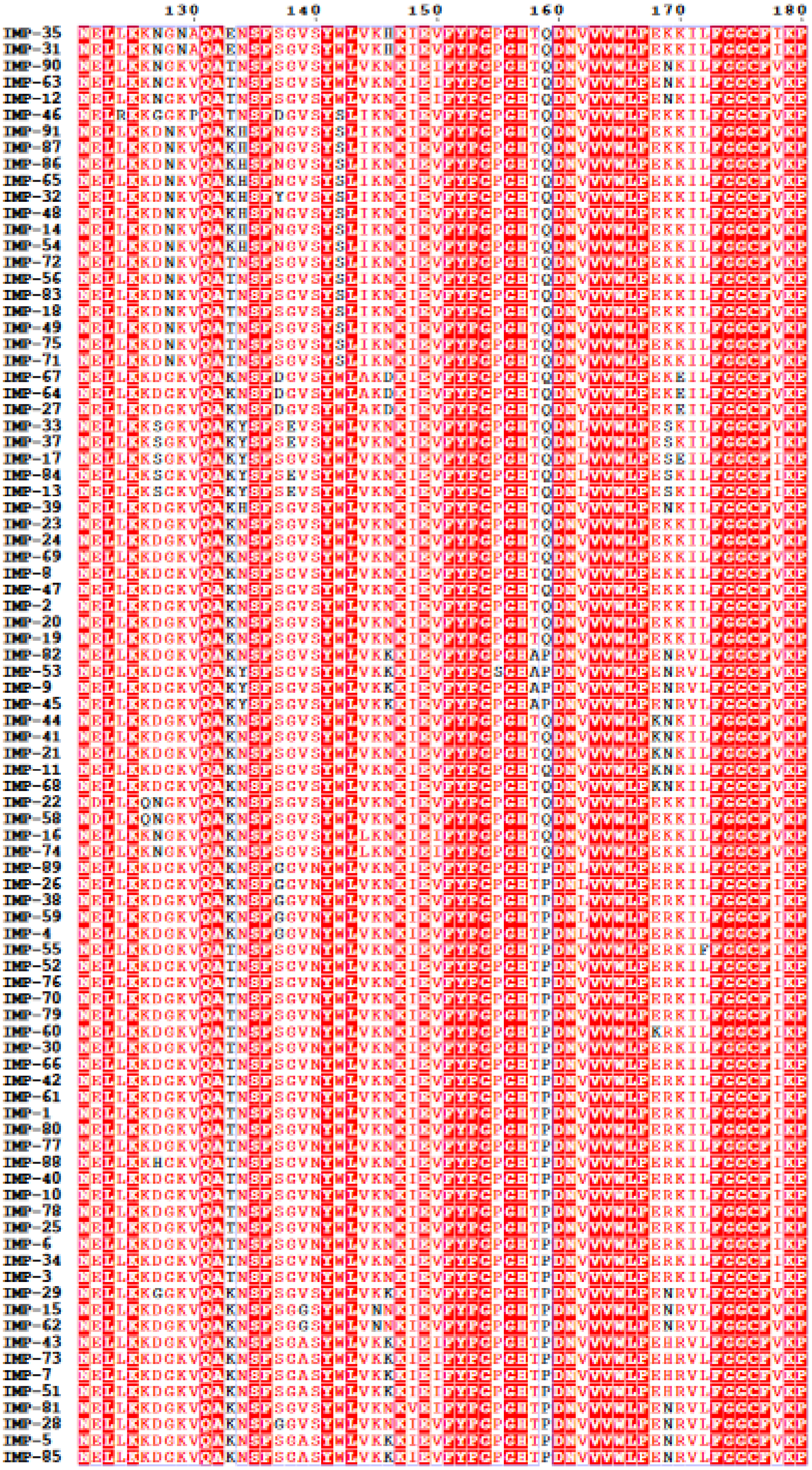
130
140
150
160
170
180
IMP-35
IMP-31
IMP-90
IMP-63
IMP-12
IMP-46
IMP-91
IMP-87
IMP-86
IMP-65
IMP-32
IMP-48
IMP-14
IMP-54
IMP-72
IMP-56
IMP-83
IMP-18
IMP-49
IMP-75
IMP-71
IMP-67
IMP-64
IMP-27
IMP-33
IMP-37
IMP-17
IMP-84
IMP-13
IMP-39
IMP-23
IMP-24
IMP-69
IMP-8
IMP-47
IMP-2
IMP-20
IMP-19
IMP-82
IMP-53
IMP-9
IMP-45
IMP-44
IMP-41
IMP-21
IMP-11
IMP-68
IMP-22
IMP-58
IMP-16
IMP-74
IMP-89
IMP-26
IMP-38
IMP-59
IMP-4
IMP-55
IMP-52
IMP-76
IMP-70
IMP-79
IMP-60
IMP-30
IMP-66
IMP-42
IMP-61
IMP-1
IMP-80
IMP-77
IMP-88
IMP-40
IMP-10
IMP-78
IMP-25
IMP-6
IMP-34
IMP-3
IMP-29
IMP-15
IMP-62
IMP-43
IMP-73
IMP-7
IMP-51
IMP-81
IMP-28
IMP-5
IMP-85

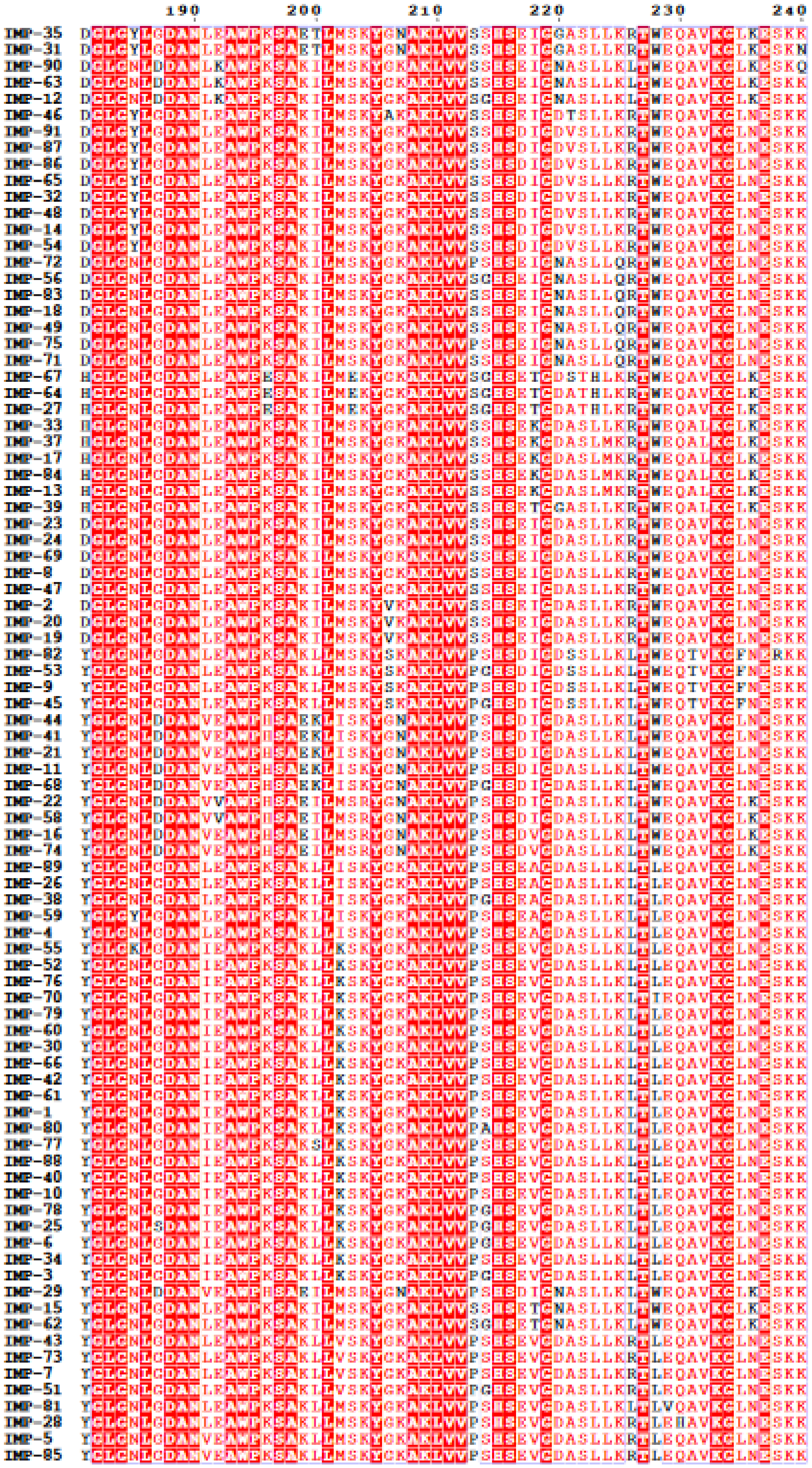

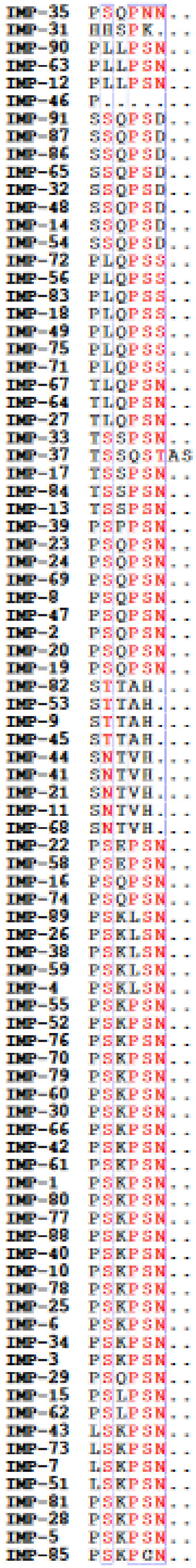

Figure S1. Multiple sequence alignment of amino acid sequence of 88 blaIMP variants.
